# Characterization of the interactions of rabbit neonatal Fc receptor (FcRn) with rabbit and human IgG isotypes

**DOI:** 10.1371/journal.pone.0185662

**Published:** 2017-09-28

**Authors:** Bence Szikora, László Hiripi, Balázs Bender, Imre Kacskovics, Attila Iliás

**Affiliations:** 1 Department of Immunology, ELTE Eötvös Loránd University, Budapest, Hungary; 2 Department of Animal Biotechnology, National Agricultural Research and Innovation Centre, Gödöllő, Hungary; 3 ImmunoGenes-ABS Ltd, Budakeszi, Hungary; Russian Academy of Medical Sciences, RUSSIAN FEDERATION

## Abstract

Despite the increasing importance of rabbit as an animal model in pharmacological studies like investigating placental transfer of therapeutic IgGs, little is known about the molecular interaction of the rabbit neonatal Fc receptor (FcRn) with rabbit and human IgG molecules. We analyzed the interactions of the rabbit and human FcRn with rabbit and human IgG isotypes using surface plasmon resonance assay. Similar to FcRn of other species, rabbit FcRn functions in pH-dependent manner, as it binds IgGs at pH 6.0, but no binding occurs at pH 7.4. We also showed that rabbit FcRn binds rabbit IgG and human IgG1 with nearly identical affinity, whereas it has stronger interactions with the other human IgG isotypes. The similar affinity of rabbit IgG and human IgG1 for rabbit FcRn was confirmed by *in vitro* FcRn-mediated recycling assay. These data verify that rabbit is an appropriate animal model for analyzing the pharmacokinetics of human therapeutic monoclonal antibodies.

## Introduction

The neonatal Fc receptor, FcRn is a heterodimer consisting of an MHC-I like α-chain and β_2_-microglobulin (β2m) [1]. FcRn plays an important role in the transcytosis of maternal IgG to the fetus and in maintaining IgG and albumin homeostasis in adult [2], as well as in antigen presentation by professional Ag presenting cells in case of Ag-IgG immune complexes [3–5].

FcRn functions in pH-dependent manner, as it binds IgG at slightly acidic pH (pH 5.5–6.0) whereas this interaction is negligible at around neutral pH (pH 7.2–7.4) [6–8]. The 2:1 FcRn:IgG binding stoichiometry, i.e. two FcRn molecules bind one IgG at independent sites was first proposed by FcRn:Fc co-crystal structures [9–11] and was further confirmed by gel filtration studies in solution [12–14], and recently, by surface plasmon resonance measurements [15]. However, FcRn can also form 1:1 complexes with the Fc region of IgG when assayed under non-equilibrium conditions [16].

In rabbit, it was found decades ago that the transfer of maternal IgG and at a lower extent, albumin occurs across the rabbit fetal yolk sac membrane (YSM) from the maternal uterine lumen to the fetus [17]. Furthermore, human IgG (hIgG) injected into the maternal circulation was also transported well to the rabbit fetus [17] indicating that rabbit FcRn (rbFcRn) binds efficiently hIgG. Low level antibody transfer could be observed during early gestation, prior to gestation day (GD) 8 due to the incomplete tight junctions of the bilaminar yolk sac membrane [18], however, no or only limited antibody transport could be detected during the period of yolk sac inversion (GD 9–13). Once inversion is completed (around GD 15), then IgG transport starts up through FcRn-mediated transcytosis, and the rate continuously increases with the progression of gestation [19–21]. Accordingly, the available data regarding the placental transfer of human therapeutic monoclonal antibodies (mAb IgGs) and Fc-containing biopharmaceuticals, similar to endogenous maternal IgG, indicate low fetal exposures until inverted yolk sac placenta is evolved, and near maternal level is reached at the end of gestation (GD 29–31) [19, 21–23].

The blood clearance of rabbit IgG (rbIgG) and hIgG was also investigated in rabbits and it was found that the half-life of rbIgG and a hIgG preparation was quite similar, around 6 and 5 days, respectively, which indicates that the FcRn-mediated salvage mechanism in rabbits works for hIgG, as well [24–26]. The similar half-lives of rbIgG and hIgG also suggest that rbFcRn binds similarly these IgGs, as IgG half-life depends on its binding affinity to FcRn [27].

Since then, it was clearly demonstrated that FcRn is highly expressed in the apical plasma membrane of the brush border’s endodermal cells of rabbit fetal yolk sac membrane (YSM) and in the placental capillary endothelial cells indicating that maternal IgG transport through the placenta is fulfilled by FcRn [28]. In addition, based on the highly conserved FcRn-IgG contact residues the pH-dependent IgG binding of FcRn was demonstrated in IgG-binding assay by Western blot using rbIgG and yolk sac lysates of rabbit fetuses [28].

Despite being an important animal model in pharmacological studies, like investigating placental transfer of therapeutic mAbs and Fc-containing biopharmaceuticals [19], the interactions of rbFcRn with rbIgG and hIgG isotypes at molecular level have not been analyzed. Therefore, we generated and purified rbFcRn and analyzed first its pH-dependent binding of rbIgG and hIgG isotypes by surface plasmon resonance assay, which provided detailed kinetic data of the interaction. Moreover, these data were further validated *in vitro* by FcRn-mediated recycling assay using rabbit macrophages.

## Materials and methods

### Amplification of soluble rbFcRn α-chain and rabbit β_2_-microglobulin (rbβ2m) cDNAs

Total RNA purification from 50 μg rabbit spleen was carried out using RNeasy Plus Kit (Qiagen, Hilden, Germany) according to manufacturer's instructions. 200 ng of DNase-treated total RNA was used for the first strand cDNA synthesis (High Capacity cDNA Reverse Transcription Kit; Life Technologies, Carlsbad, CA, USA). Negative control reverse transcription reactions were conducted to confirm no genomic DNA contamination in the RNA preparation.

The spleen cDNA sample was used as PCR templates to amplify the full-length coding region of rbβ2m and the truncated, soluble FcRn α-chain (lacking the transmembrane and the cytoplasmic domains) cDNAs, respectively, using gene specific primers. Specific oligonucleotides were used to amplify rbFcRn α-chain cDNA (forward: 5’- ATCAGAATTCCCTATAAATATGGGGCGCCCCCGGCTT -3’; reverse: 5’- ATCAGAATTCCTAATGATGATGATGATGATGACGACCTTCGATCAGCGCCACCGACAGCGGCT—3’). EcoRI linker and insect Kozak sequence (5’- CCTATAAATATG -3’) was also added to the 5’ end of the forward oligo, while EcoRI linker, 6xHis tag and Factor Xa recognition sequence (5’- ACGACCTTCGAT -3’) was added to the 5’ part of the reverse oligonucleotide. rbβ2m was amplified with specific oligonucleotides (forward: 5’- ATCAGGATCCCCTATAAATATGTCGCGCTCCGTCTTGGG -3’; reverse: 5’- ATCAGGATCCTGTTGATTAGTAATCTCGAT -3’). Both oligonucleotide primers contained BamHI linker sequence, furthermore the forward oligo harbored insect Kozak linker sequence, as well.

PCR reactions were performed in a 20-μl reaction mix (RedTaq ReadyMix; Sigma-Aldrich, St. Louis, MO, USA) with the condition of a 5 min denaturation at 94°C followed by 32 cycles of 94°C for 30 sec, 59°C for 45 sec, and 72°C for 30 sec, and a final extension at 72°C for 3 min. DNA fragments obtained by RT-PCR were purified from agarose gels using a QIAquick Gel extraction kit (Qiagen, Hilden, Germany) and sequenced directly.

### Construction of the baculovirus expression vector

To co-express soluble rbFcRn and rbβ2m in Sf9 cells the cDNA of rbFcRn α-chain was inserted into the EcoRI site in the baculovirus transfer vector, pAcUW51 under the control of the p10 promoter. The rbβ2m cDNA was then inserted into the BamHI site under the control of the polyhedrin promoter of pAcUW51 containing rbFcRn α-chain cDNA. Orientation of both inserts was verified by restriction analysis and by sequencing.

### Expression and purification of soluble rbFcRn

Recombinant baculoviruses carrying the cDNAs of the soluble rbFcRn α-chain and rbβ2m were generated by using the BaculoGold Transfection Kit (BD Biosciences Pharmingen, Franklin Lakes, NJ, USA) according to the manufacturer’s instructions. Then, Sf9 cells were cultured and infected by recombinant viruses. Recombinant rbFcRn was purified from supernatants of virus infected cells by using Ni-NTA chromatography (His-Select Nickel Affinity Gel, Sigma-Aldrich, St. Louis, MO, USA) according to the manufacturer’s protocol. Expression level and purity of the recombinant protein was confirmed by 15% SDS-PAGE followed by either Coomassie staining or Western blotting. Mouse anti-His tag antibody (AbD Serotec, Kidlington, UK) as the primary (500x) and HRP-conjugated goat anti-mouse IgG (Southern Biotech, Birmingham, AL, USA) as the secondary antibody (10.000x) were used for immunodetection. The yield of the rbFcRn expression was approximately 5 mg/l (5 mg purified protein per 1 liter cell culture supernatant).

### Size-exclusion chromatography

The aggregations of the purified FcRn samples were analyzed by size-exclusion chromatography (SEC). Agilent-1100 chromatographic system (Agilent Technologies, Santa Clara, CA, USA) equipped with UV detector was employed with a Yarra SEC-3000 column (l: 300mm, id.: 4,6mm; Phenomenex, Torrance, CA, USA). Mobile phase composition was 50 mM phosphate buffer, pH 6.8 supplemented with 0.3 M potassium chloride and the volumetric rate of mobile phase was 0.35 ml/minutes. Column temperature was 30°C.

Samples were diluted to 1 mg/ml protein concentrations in PBS-T buffer, pH 7.4 (10 mM Na-phosphate, 150 mM NaCl, 0.005% Tween 20). 5 μl sample solutions were injected for analysis and monitored at 215 nm. Molecular weight of the detected sample components was calculated using calibration curves. A molecular weight standard solution (ALO-3042, Phenomenex, Torrance, CA, USA) that contains reference proteins from 17 kDa to 670 kDa and 1 mg/l BSA solution as an additional reference was used for establishing calibration curves and for testing the resolution of chromatographic system. The calibration curves were linear in the range studied. The detected components were identified according to the molecular weight calculated and the results were expressed as the purity (percentage area) of the principal (FcRn) component.

### Surface plasmon resonance (SPR) measurements

The preparation of the GLC sensor chip and the immobilization procedure by amine coupling were performed according to the manufacturer’s instructions. Briefly, GLC chip was inserted to the ProteOn XPR36 biosensor instrument (Bio-Rad, Hercules, CA, USA), and was initialized by 50% glycerol. The immobilization procedure was carried out in the vertical orientation at a flow rate of 30 μl/min in PBS-T buffer, pH 7.4 (10 mM Na-phosphate, 150 mM NaCl, 0.005% Tween 20) at 25°C. Thereafter, 0.2 M EDAC and 0.05 M sulfo-NHS were mixed in 1:1 ratio and the solution was injected immediately with 2 min of contact time in order to activate the chip surface. This was followed by an immediate injection of 150 μl of ligand solutions containing 10 μg/ml of rbFcRn and human FcRn (the latter, similar to our rbFcRn, is a soluble form of hFcRn, which was a kind gift from Sally E. Ward, UT Southwestern Medical Center, Dallas, TX, USA), or 2 μg/ml of rbIgG (ATG-Fresenius, Fresenius Biotech, Bad Homburg, Germany) and hIgG1 (Omalizumab, Genentech, San Francisco, CA, USA), respectively, in 10 mM Na-acetate buffer, pH 5.0, for 5 min. For detecting background responses, a control channel without any protein was prepared following the same procedure. Finally, the remaining activated carboxyl groups on the chip surface were neutralized by the injection of 150 μl of 1 M ethanolamine-HCl, pH 8.5 for 4 min. This resulted in the immobilization of approximately 1200 and 1600 RU in case of rbFcRn and hFcRn, respectively, and concerning rbIgG and hIgG1 the immobilization levels were approximately 1900 and 2100 RU, respectively.

Binding experiments were performed with a continuous flow (100 μl/min) of PBS-T buffer, at pH 6.0 or pH 7.4 allowing for a contact time of 2 min at 25°C. Five different concentrations (8.34, 16.7, 33.3, 66.7 and 133 nM) of rbIgG (ATG-Fresenius, Fresenius Biotech, Bad Homburg, Germany) and hIgG isotypes (hIgG1: Omalizumab, Genentech, San Francisco, CA, USA; hIgG2, hIgG3, hIgG4: Abcam, Cambridge, UK) were injected in the horizontal orientation of channels over immobilized FcRn molecules, respectively, and running buffer, PBS-T was injected simultaneously to the first channel. Similarly, when IgG molecules were immobilized on the chip surface, five different concentrations (37.5, 75, 150, 300 and 600 nM) of rbFcRn and hFcRn were injected. After the measurement, the chip surface was regenerated by a 30 sec injection of 0.1 M Tris, pH 8.0 to remove any remaining analyte.

All binding sensorgrams were collected and processed using the integrated ProteOn Manager software, version 3.1.0.6 (Bio-Rad, Hercules, CA, USA). The curves were corrected by subtracting the non-specific binding responses obtained from control channel. Then, the kinetic analyses were performed by the BIAevaluation software, version 4.1 (Biacore, GE Healthcare, Little Chalfont, United Kingdom). Binding curves were fit to either Langmuir 1:1 interaction or heterogeneous ligand model supposing that there are two classes of non-interacting binding sites. The equilibrium dissociation constants or affinity constants were calculated from the directly estimated association and dissociation rate constants (Langmuir model: K_D_ = k_d_/k_a_ or heterogeneous ligand model: K_D1_ = k_d1_/k_a1_ and K_D2_ = k_d2_/k_a2_) and in case of the latter model the percentage of the total response according to each class of binding sites (f_1_ and f_2_) were also estimated.

### Fluorescent IgG preparation and FcRn-mediated recycling assay in rabbit macrophages

rbIgG and hIgG1 were conjugated to Alexa Fluor 488 (A488) using the Alexa Fluor 488 carboxyl acid succinimidyl ester labeling kit (Molecular Probes, Thermo Fisher Scientific, Waltham, MA, USA) according to the manufacturer’s instructions. Labeled proteins were separated from free dye using Vivaspin 2 centrifugal concentrator (10,000 MWCO PES; Sartorius, Dublin, Ireland). Protein concentration, and degree of labeling were determined by spectrophotometry using Nanodrop 2000 instrument (hIgG1: 5.5 mg/ml, conjugation rate: 0.8, rbIgG: 5.9 mg/ml, conjugation rate: 1.9).

Two females of New Zealand White rabbit (S&K-LAP Ltd, Kartal, Hungary) were used in this study. Animals were kept under standard light-dark cycle (06.00–18.00 h) at 19°C with food and water available *ad libitum* and caged separately. All the treatments of animals (rabbits) in this research followed by the guideline of the Institutional Animal Care and Ethics Committee at ImmunoGenes-ABS Ltd that operated in accordance with permission XIV-I-001/2086-4/2012 issued by the Food Chain Safety and Animal Health Directorate of the Government Office of Pest County, Hungary. All surgery was performed under ketamine/xylazine anesthesia. The method used for euthanasia: concussion under anesthesia and all efforts were made to minimize suffering.

Rabbit macrophages were isolated from the peritoneum of a New Zealand White rabbit 3 days after i.p. injection of 40 ml 3% Brewer thioglycol medium based on a standard protocol [29]. Red blood cells were lysed in hypotonic buffer (45 mM NH_4_Cl, 2.5 mM KHCO_3_, 0.05 mM Na_2_EDTA, pH 7.2). 1 μ-slide 8-well plates (Ibidi, Martinsried, Germany) were coated with 100 ng/ml fibronectin, and 3*10^5^ cells were added in 300 μl RPMI-1640 medium supplemented with 10% fetal bovine serum and allowed to adhere for O/N at 37°C in a CO_2_ incubator. Then, the non-adherent cells were washed out and the cells were pulsed by adding 300 μl 20 μg/ml fluorescently labeled (A488) rbIgG or hIgG1 to the cells for 20 minutes in Hank’s buffer, pH 6.0 (143 mM NaCl, 1 mM Na_2_SO_4_, 5 mM KCl, 1 mM NaH_2_PO_4_, 0.5 mM MgCl_2_, 1 mM CaCl_2_, 5 mM glucose, 10 mM HEPES). After washing twice, the chase was started by replacing buffer to Hank’s buffer, pH 7.4 lacking fluorescent probes, and the amount of labeled antibodies accumulated in the cells was visualized at 0 and after 30 minutes by Olympus Fluoview500 inverted Laser Scanning Confocal Microscope (Olympus-Europe, Hamburg, Germany; at 20x magnification). For image processing and evaluation Fiji software [30] was used. Based on the bright field image a region of interest was drawn around individual well-adherent cells and due to the different labeling efficiency of rbIgG and hIgG1 the median pixel intensity was recorded. Minimum of 30 cells were analyzed from each sample, but cells with large amount of saturated pixels were eliminated from analysis. The fluorescence signal in each image plane was corrected for background (by subtracting the median fluorescence intensity of five different cell-free area). Paired two-sample t-test was used for the statistical analyses.

## Results and discussion

### Amino acid residues in FcRn-IgG interaction

The truncated form of the rbFcRn α-chain and the rbβ2m were PCR cloned. The cDNA sequences showed 100% identity with the published rbFcRn α-chain sequence [28] and the annotated rbβ2m sequence (XM_008269078). At protein level, Asn was found at position 95, as well as at position 97 in the only published sequence of rbβ2m [31], whereas NCBI reference sequence analysis (XP_008267300.1) and our cDNA clone (the sequence was deposited into GenBank: KT151665) predicted Asp at both positions.

**Fig 1:**
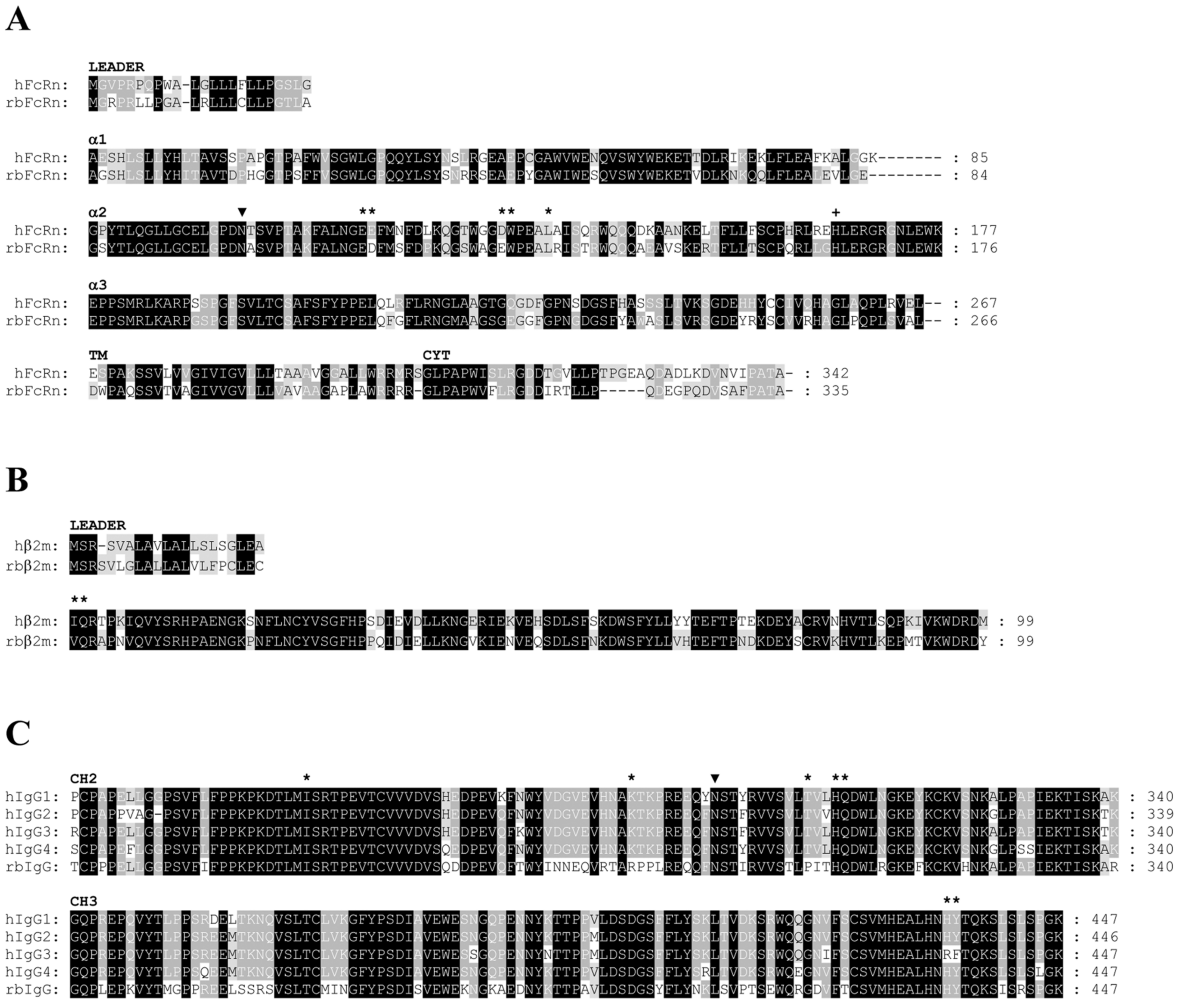
Domain-by-domain alignment of the amino acid sequences for human and rabbit variants of FcRn α-chain, β2m and IgG isotypes. Structural and functional features are highlighted in the amino acid sequences of hFcRn and rbFcRn α-chains (Panel A), rabbit and human β2m (Panel B) and CH2, as well as CH3 domains of human and rabbit IgG isotypes (Panel C). Consensus residues are assigned based on the number of occurrences of the character in the column, emphasizing the degree of conservation. The higher the conservation in a column the darker the background of the character [32]. Potential N-linked glycosylation sites are indicated by filled triangle. Residues at the interface between FcRn and Fc region of IgG based on a crystallography analysis of a rat FcRn-heterodimeric Fc complex [10] are labelled with asterisks. Conserved His at position 166 in FcRn α-chain sequences (Panel A) is considered to bind to albumin [33] is indicated by a + sign. Numbering is based on the hFcRn α-chain sequence.

The sequence comparisons show high identity between human (GenBank: ARO48560) and rabbit (Genbank: AEN74950) [28] variants of FcRn α-chain (72%), human (GenBank: CAG33347) and rabbit (GenBank: KT151665) β2m (74%), as well as human (hIgG1 GenBank: P01857, hIgG2 GenBank: P01859, hIgG3 GenBank: P01860, hIgG4 GenBank: P01861) and rabbit IgG (GenBank: P01870) isotypes (69–71%) querying the NCBI Reference Sequences database with Blastp. There is one potential N-linked glycosylation sites (N-X-S or N-X-T; where X is any amino acid except proline) at position 102 in both FcRn α-chain sequences (numbering is based on the human sequence) and another one at position 297 in CH2 domain of all human and rabbit IgG isotype sequences (numbering is based on the hIgG1 sequence) (Fig 1).

Focusing on the important residues of the FcRn-IgG interaction in the hFcRn and rbFcRn α-chain sequences based on a crystallography analysis of a rat FcRn-heterodimeric Fc complex [10], we found that these amino acids are highly conserved between the two species. Only two conservative substitutions can be observed, Asp instead of Glu at position 116 and Glu instead of Asp at position 130 (amino acid numbering follows the human sequence) in rbFcRn α-chain comparing with human sequence (Table 1).

**Table 1:** FcRn and IgG residues known to be involved in FcRn–IgG interaction.

α-chain residue	Human	Rabbit	Fc residue	Human IgG				Rabbit IgG
				1	2	3	4	
115	E	E	310	H	H	H	H	H
116	E	D	311	Q	Q	Q	Q	Q
130	D	E	435	H	H	R	H	H
131	W	W	253	I	I	I	I	I
135	L	L	436	Y	Y	F	Y	Y
β2m residue								
1	I	V	307	T	T	T	T	P
2	Q	Q	288	K	K	K	K	R

Numbering is based on the hFcRn, hβ2m and the hIgG1 sequences, respectively.

Between the two residues in rbβ2m sequence playing role in the interaction with IgG one is conserved (Gln2 in both species), while Ile at position 1 in the hβ2m is replaced by Val in the rabbit sequence which is considered to be also a conservative substitution (Table 1).

Analyzing the sequences of human and rabbit IgG isotypes no amino acid changes can be observed in the sequences of rbIgG, hIgG1, hIgG2 and hIgG4 concerning the residues involved in the interaction with FcRn α-chain, moreover, only two residues vary in hIgG3, Arg can be found at position 435 instead of His, and Phe at postion 436 instead of Tyr (Table 1).

However, both residues are replaced in rbIgG at positions being important in the interaction with β2m, all human IgG isotypes contains Thr and Lys at positions 307 and 288, while Pro and Arg occur in these positions of the rbIgG sequence, respectively.

Altogether, we can conclude that except T307P replacement in the rbIgG sequence all the contact residues being involved in FcRn-IgG interaction are conserved between human and rabbit variants suggesting similarities in their pH-dependent mechanism of action.

### Expression of the soluble form of rbFcRn

**Fig 2:**
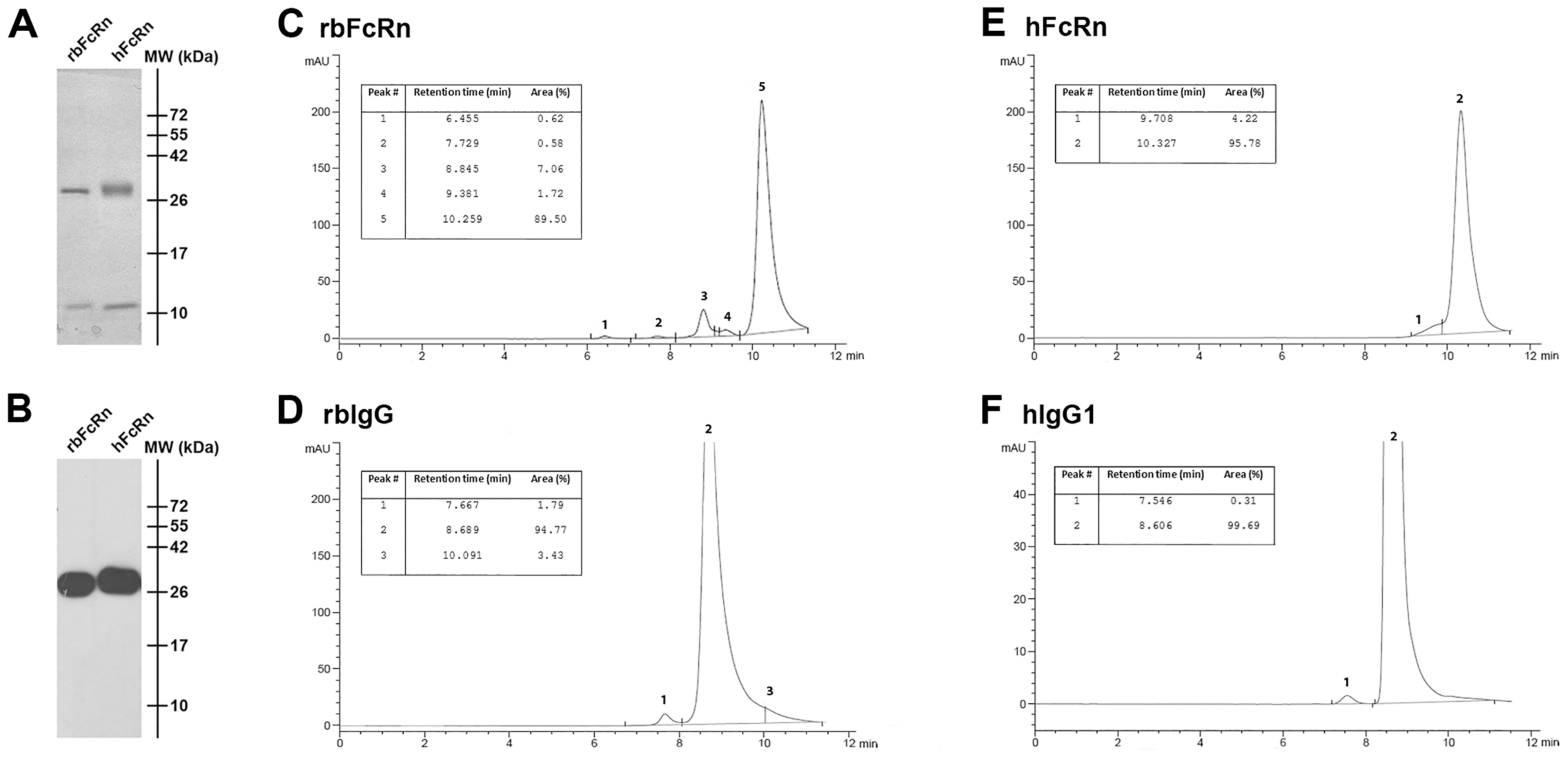
Expression and SEC analysis of the soluble form of rbFcRn purified from Sf9 cell supernatant. The expression of rbFcRn was detected by 15% SDS-PAGE followed by Coomassie staining. The rbFcRn α-chain, as well as the rbβ2m was observed around 28 and 12 kDa, respectively (Panel A). By using anti-His tag monoclonal Ab in Western blot experiments only the rbFcRn α-chain containing 6xHis-tag can be detected (Panel B). As a control, soluble hFcRn was used in both experiments. The purity of rbFcRn (Panel C) and rbIgG (Panel D), as well as hFcRn (Panel E) and hIgG1 (Panel F) samples was verified by size-exclusion chromatography.

The cDNAs of truncated rbFcRn α-chain and rbβ2m were co-expressed in Sf9 insect cells and the self-assembled soluble recombinant rbFcRn was purified from the cell supernatants using Ni-NTA chromatography. The expression level was confirmed by SDS-PAGE visualized by Coomassie staining where two bands appeared, an approximately 28 kDa band referring to FcRn α-chain and another about 12 kDa band to the β2m, similarly to the soluble hFcRn (Fig 2A). By using anti-His-tag-specific monoclonal antibody in Western blot experiments the rbFcRn and hFcRn α-chains containing 6xHis-tag could be detected around 28 kDa (Fig 2B). The purity of the FcRn molecules, as well as the rbIgG and hIgG samples was analyzed by SEC which revealed that the purity of hIgG1 and rbIgG, as well as hFcRn was >95%, while the rbFcRn preparation was around 90%, but it did not contain any higher-order aggregates (Fig 2C–2F). Some residual molecule (at 8.845 and 9.381 mins of retention time, altogether approximately 9%), potentially bovine serum albumin (BSA) remained in the preparation after purification (Fig 2C), as these fractions ran equivalently with BSA in the molecular weight standard and the ratio of these components could be increased by adding extra BSA. However, as BSA does not react with IgGs, it did not influence our further analyses.

### pH-dependence and isotype-specific differences of IgG binding

Detailed kinetic data of the interaction of human, cynomolgus monkey, bovine, rat and mouse FcRn with IgG isotypes from different species are available [11, 15, 34–37], however, to date no data has been reported concerning the kinetics of the rbFcRn interaction with rbIgG and hIgG isotypes.

FcRn experts apply two different SPR experimental set-ups, either FcRn or IgG molecules are immobilized on the sensor chip surface. Accordingly, depending on which molecule is immobilized on the surface the evaluation methods, the determined kinetic parameters can be different. When FcRn is immobilized and IgG molecules are injected as analytes, the FcRn-IgG interaction can be characterized only by complex kinetics supposing two classes of non-interacting binding sites described by heterogeneous ligand model [11, 13, 35, 38–40]. In opposing assay orientation, when IgG is coupled to the surface and FcRn molecules are injected over it, the interaction can be described by simple Langmuir 1:1 binding model [13, 15, 37]. Examples for both set-ups can be found in the literature, however, to date there is no consensus concerning the accepted orientation, immobilization and evaluation methods [15, 37]. Therefore, the IgG binding of rbFcRn was analyzed using both experimental set-ups in our SPR experiments, either rbFcRn and hFcRn or rbIgG and hIgG1 molecules were immobilized.

#### Immobilization of FcRn

**Fig 3:**
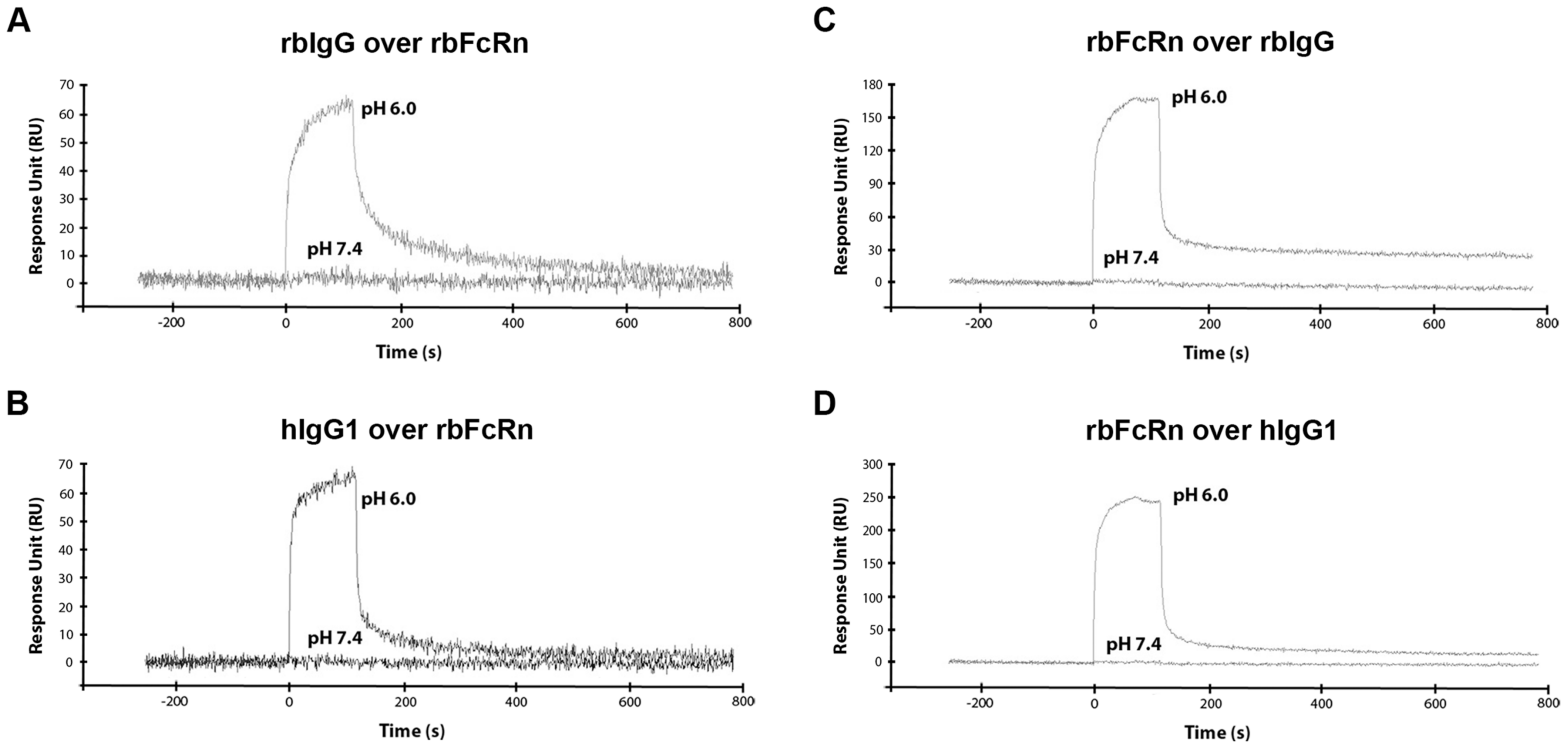
pH-dependent binding of rbIgG and hIgG1 by rbFcRn. Soluble rbFcRn (Panel A and B), as well as rbIgG (Panel C) and hIgG1 (Panel D) were immobilized on a GLC chip at densities of 1200, 1900 and 2100 RU, respectively. rbIgG (Panel A) and hIgG1 (Panel B) at 133 nM, while soluble rbFcRn (Panel C and D) at 600 nM concentration in PBS-T buffer were injected at pH 6.0, as well as at pH 7.4 and interactions were monitored at 25°C. The curves were corrected by subtracting the non-specific binding responses obtained from control channel (n = 2–3, one representative figure is shown in each cases).

Soluble forms of rbFcRn and hFcRn were covalently immobilized onto the chip surface, and dilutions of rbIgG and hIgG isotypes were injected over them. Our results demonstrated that rbFcRn, similarly to other FcRn orthologues binds IgG in strictly pH-dependent manner, as it interacts with rbIgG, as well as hIgG isotypes at pH 6.0 (binding of rbIgG and hIgG1 at 133 nM concentration are shown on Fig 3A and 3B), while no binding could be detected at pH 7.4 using rbIgG (Fig 3A), hIgG1 (Fig 3B), hIgG2, hIgG3 and hIgG4. hFcRn was used as a control, and we measured very similar binding kinetics in the presence of either rbIgG or hIgG isotypes at pH 6.0 and also at pH 7.4.

In an earlier study, it was suggested that in rabbit, binding and selective transcytosis of IgG through rabbit visceral yolk sac (VYS) did not require acidic compartment and thus, rbFcRn binds IgG in pH-independent manner [41]. In contrast to this observation our previous study demonstrated that the binding of IgG to rbFcRn was pH-dependent using an *in vitro* IgG-binding assay with yolk sac lysates of rabbit fetuses [28]. In accordance with the latter finding our SPR measurements clearly demonstrated that rbFcRn interacts with rbIgG and hIgGs at pH 6.0 and no binding occurs at pH 7.4.

**Fig 4:**
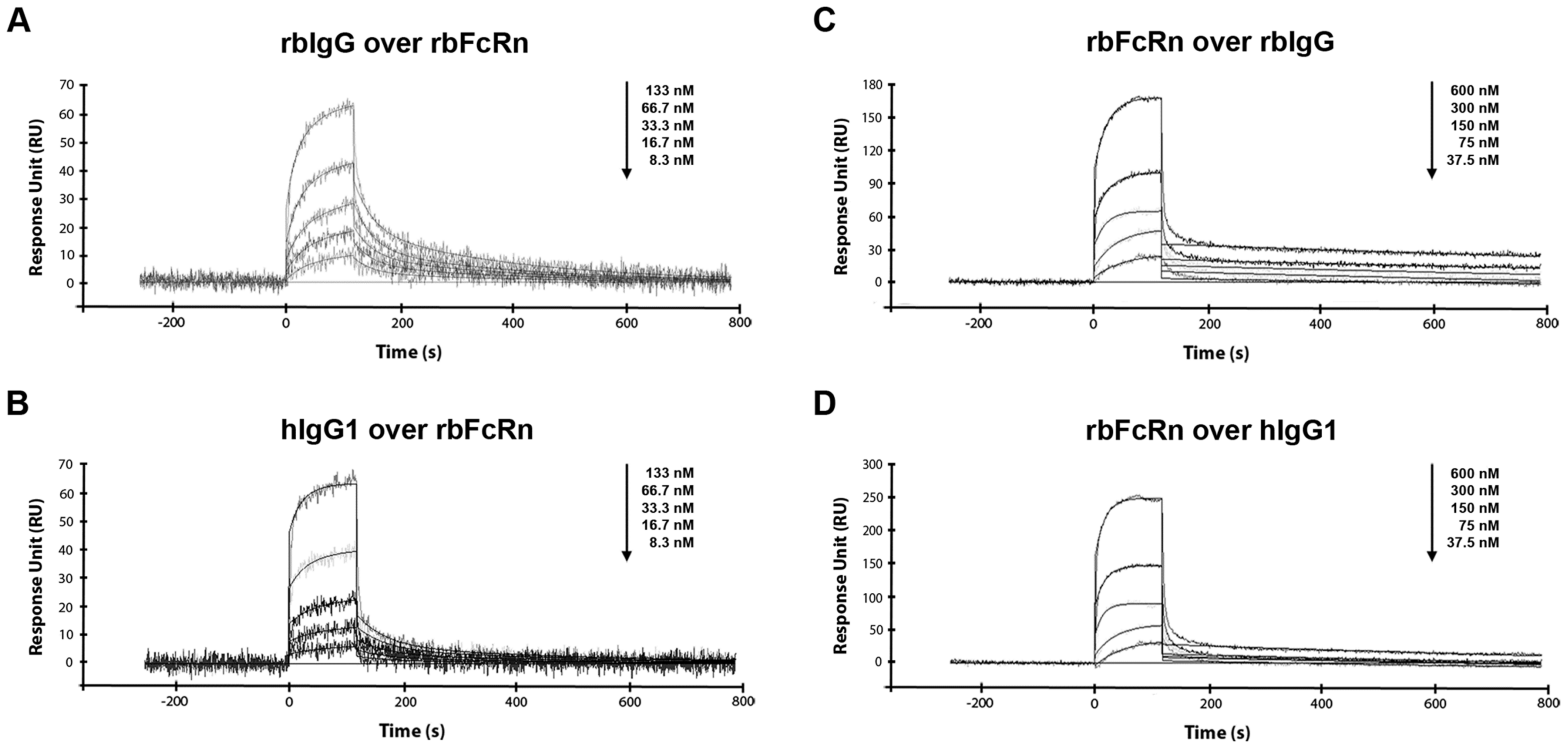
Sensorgrams for the interaction of soluble rbFcRn with rbIgG and hIgG1. Varying rbIgG and hIgG1 (8.34 to 133 nM) or soluble rbFcRn (37.5 to 600 nM) concentrations were injected over immobilized soluble rbFcRn or rbIgG and hIgG1, respectively, on the chip surface. In both experimental set-ups, the interactions were monitored at pH 6.0 and at 25°C and the curves were corrected by subtracting the non-specific binding responses obtained from control channel. Heterogeneous ligand model (Panel A and B) or Langmuir 1:1 binding model (Panel C and D) was fitted to the sensorgrams by grouped analysis using BIAevaluation software (n = 2–3, one representative figure is shown in each cases).

Injecting a series of concentrations of rbIgG and hIgG isotypes over rbFcRn and hFcRn immobilized on the chip surface, the kinetic parameters of IgG binding of both rbFcRn and hFcRn could be determined. Fits of sensorgrams were performed using the heterogeneous ligand model postulating two or more populations of non-interacting binding sites [11, 13, 38] (Fig 4A and 4B).

By means of this model, a high affinity constant, K_D1_ when two FcRn molecules bind one IgG and a low affinity constant, K_D2_ referring to 1:1 FcRn:IgG stoichiometry can be distinguished (Table 2).

**Table 2:** Kinetic parameters of the interaction of immobilized rbFcRn and hFcRn with rbIgG and hIgG isotypes at pH 6.0.

	rbFcRn				hFcRn			
	K_D1_ (M) x 10^−9^	f1 (%)	K_D2_ (M) x 10^−9^	f2 (%)	K_D1_ (M) x 10^−9^	f1 (%)	K_D2_ (M) x 10^−9^	f2 (%)
rbIgG	20.3 ± 2.21	68.4	49.3 ± 1.78	31.6	6.58 ± 0.12	77.0	15.9 ± 0.27	23.0
hIgG1	12.9 ± 1.83	28.8	71.9 ± 15.7	71.2	9.99 ± 0,43	42.4	57.2 ± 3.05	57.6
hIgG2	7.46 ± 1.41	84.3	106 ± 31.5	15.7	5.43 ± 0.26	89.2	34.7 ± 0.68	10.8
hIgG3	2.57 ± 0.52	49.9	59.2 ± 7.25	50.1	6.06 ± 0.25	74.8	17.4 ± 0.50	25.2
hIgG4	3.86 ± 0.79	89.8	53.5 ± 20.0	30.5	2.36 ± 0.14	92.1	20.2 ± 1.01	7.9

The K_D_ values (mean ± S.E.) were calculated from the estimated association and dissociation rate constants (K_D1_ = k_d1_/k_a1_) and (K_D2_ = k_d2_/k_a2_) using heterogeneous ligand model during the fitting procedure (n = 2–3).

Comparing the high affinity constants (K_D1_) of the interactions we found that rbFcRn binds hIgG3 and hIgG4 with the highest affinity (2.57 and 3.86 nM, respectively), hIgG2 with a slightly lower affinity, 7.46 nM, which interactions were apparently stronger than that of hIgG1 (12.9 nM) and rbIgG (20.3 nM). The low affinity constants (K_D2_) of rbIgG, hIgG4 and hIgG3 were almost identical, 49.3, 53.5 and 59.2 nM, respectively, while hIgG1 and hIgG2 showed weaker (71.9 and 106 nM, respectively) interactions.

As our SPR data revealed, rbFcRn showed almost identical K_D_ values for hIgG1 and slightly higher affinity for other hIgG isotypes than for rbIgG. The stronger interaction with IgG from another species is not a unique feature of rbFcRn, because similar cross-species binding differences were observed concerning mouse, as well as bovine FcRn, which showed also higher affinity for hIgG1 than for their own, mouse [36, 40, 42, 43] and bovine IgG isotypes [35], respectively.

Being aware of kinetic data of the interaction of rbFcRn with hIgG isotypes it would be interesting to interpret the placental transport of human therapeutic mAbs and Fc-containing biopharmaceuticals performed previously in rabbits. Comparing the fetal/maternal serum concentration ratio of IgG1 and IgG4 mAbs and Fc-fusion proteins provided with similar doses and similar blood sampling time (GD 19–20) in rabbits, it was found that the placental transfer of an IgG4 mAb and an Fc-fusion protein across the YSM was more effective, 63- and 3.5-fold higher, respectively, than that of IgG1 isotypes [19]. This observation is correlated well with our affinity data determined by SPR, where rbFcRn showed approximately 3-fold comparing the high, K_D1_ (3.86 nM vs. 12.9 nM), and almost the same affinity for IgG4 than for IgG1 comparing the low affinity constant, K_D2_ (53.5 nM vs. 71.9 nM).

In another study, it was found that a fully hIgG2 mAb was also effectively transported through the rabbit placenta during early organogenesis (GD 10 and 13), however, this transport, similarly to rat and cynomolgus monkey at this similar stage, was low [44]. In support of this, it was found that FcRn mRNA appears in rabbit embryos at GD 6 and the expression continuously increased until GD 13.5 [28]. Our SPR results showed that rbFcRn binds hIgG2 efficiently, since 3-fold difference could be observed in high affinity constants (7.46 nM for hIgG2 and 20.3 nM for rbIgG), however, almost the opposite interaction was measured concerning the low affinity constants (106 nM for hIgG2 and 49.3 nM for rbIgG).

Analyzing the high affinity interactions of hFcRn with rbIgG and hIgG isotypes only little difference could be seen in affinities: hIgG4 has the highest affinity (2.36 nM), rbIgG, hIgG2 and hIgG3 bind similarly to hFcRn (6.58, 5.43 and 6.06 nM, respectively), whereas the interaction is slightly weaker concerning hIgG1 (9.99 nM). The low affinity constants of rbIgG and hIgG3 showed the lowest (15.9 and 17.4 nM), hIgG1 showed the highest value (57.2 nM), and the other K_D2_ values were in between (20.2 nM for hIgG4 and 34.7 nM for hIgG2). These data agree well with K_D_ values obtained by others also with hIgG isotypes and immobilized hFcRn in SPR measurements [11, 38, 39].

Polyclonal rbIgGs so-called anti-thymocyte globulin (ATG) preparations (like ATG-Fresenius by Fresenius Biotech, Bad Homburg, Germany and Thymoglobulin by Sanofi Genzyme, Cambridge, MA, USA) are extensively used as immunosuppressant agents in clinical applications, mainly in the field of human transplantation [45]. Our SPR results revealed that rbIgG binds to hFcRn as effective as hIgG isotypes, therefore it can successfully compete with endogenous IgG and can be protected from the degradation by FcRn in human which can explain the success of rabbit anti-thymocyte globulin therapy.

#### Immobilization of IgG

As hIgG1 is used mostly in therapeutic mAbs or Fc-fusion proteins, we have focused this human isotype along rbIgG in the next experiment. When rbIgG and hIgG1 were immobilized on the chip surface, and rbFcRn and hFcRn were injected over, similar results were obtained what was observed at reverse orientation, namely, rbFcRn binds rbIgG and hIgG1 in a strictly pH-dependent manner (Fig 3C and 3D).

Next, rbFcRn and hFcRn were injected at varying concentrations (37.5 to 600 nM) over the surface in order to determine the kinetic parameters of the interaction. This experimental set-up gives the possibility to apply the Langmuir 1:1 binding model in kinetic analyses instead of complex kinetics e.g heterogeneous ligand model [13, 15, 37] (Fig 4C and 4D).

Kinetic parameters were determined and data showed that rbFcRn-rbIgG and rbFcRn-hIgG1 interactions have nearly identical affinity (1.48 and 1.19 nM, respectively), while hFcRn binds rbIgG with a slightly lower (2.46 nM) and hIgG1 with even lowest affinity (4.30 nM) (Table 3).

**Table 3:** Kinetic parameters of the interaction of immobilized rbIgG and hIgG1 with rbFcRn and hFcRn at pH 6.0.

	rbFcRn			hFcRn		
	k_a_ (M^-1^s^-1^) x 10^5^	k_d_ (s^-1^) x 10^−4^	K_D_ (M) x 10^−9^	k_a_ (M^-1^s^-1^) x 10^5^	k_d_ (s^-1^) x 10^−4^	K_D_ (M) x 10^−9^
rbIgG	2.67 ± 0.17	3.94 ± 0.13	1.48 ± 0.14	3.23 ± 0.31	7.92 ± 0.34	2.46 ± 0.34
hIgG_1_	3.27 ± 0.16	3.90 ± 0.14	1.19 ± 0.10	1.38 ± 0.10	5.92 ± 0.13	4.30 ± 0.41

The K_D_ values (mean ± S.E.) were calculated from the estimated association and dissociation rate constants (K_D_ = k_d_/k_a_) using Langmuir 1:1 binding model during the fitting procedure (n = 2–3).

These results are in accordance with the parameters obtained at reverse orientation, namely rbFcRn binds hIgG1 similarly to rbIgG. This explains why FcRn-mediated salvage mechanism and maternal IgG transport in rabbits function efficiently for intravenously injected hIgG1, as previously described [17]. In addition, this transplacental transport is supposed to be proportional to the amount of hIgG1 found in the serum. This is important assumption in regard to rabbits that are widely used as animal model in transplacental studies of human therapeutic monoclonal antibodies (IgG).

In order to validate our SPR results, the interaction of rbFcRn with rbIgG and hIgG1 was investigated *in vitro* by FcRn-mediated recycling assay using rabbit macrophages.

### FcRn-mediated recycling assay using rabbit macrophages

FcRn-mediated IgG protection primarily takes place in capillary endothelial cells and also in hemopoietically-derived phagocytic cells, like macrophages [46]. Accordingly, significant FcRn expression could be detected in bone-marrow-derived macrophages in mice [4, 46], as well as in peritoneal macrophages in rabbits [28].

Rabbit macrophages isolated from the peritoneum of a New Zealand White rabbit were then used for FcRn-mediated recycling experiments. Isolated cells were plated and allowed to adhere. After removal of non-adherent cells the pulse step was started at pH 6.0 by adding fluorescently labeled rbIgG or hIgG1 to the cells which lasted for 20 minutes. Pulsing at slightly acidic pH, due to FcRn-IgG interaction cells can only take up IgG via fluid-phase pinocytosis, Fcγ-receptor- or perhaps even FcRn-mediated process, but IgG cannot be released from the cell to the extracellular space due to the pH dependent binding. Then, the cells were chased for 30 minutes by changing pH to 7.4 by replacing medium. The amount of labeled antibodies accumulated in the cells after 0 and 30 minutes of chase was calculated by determining the median value of fluorescent intensity of each cell.

**Fig 5:**
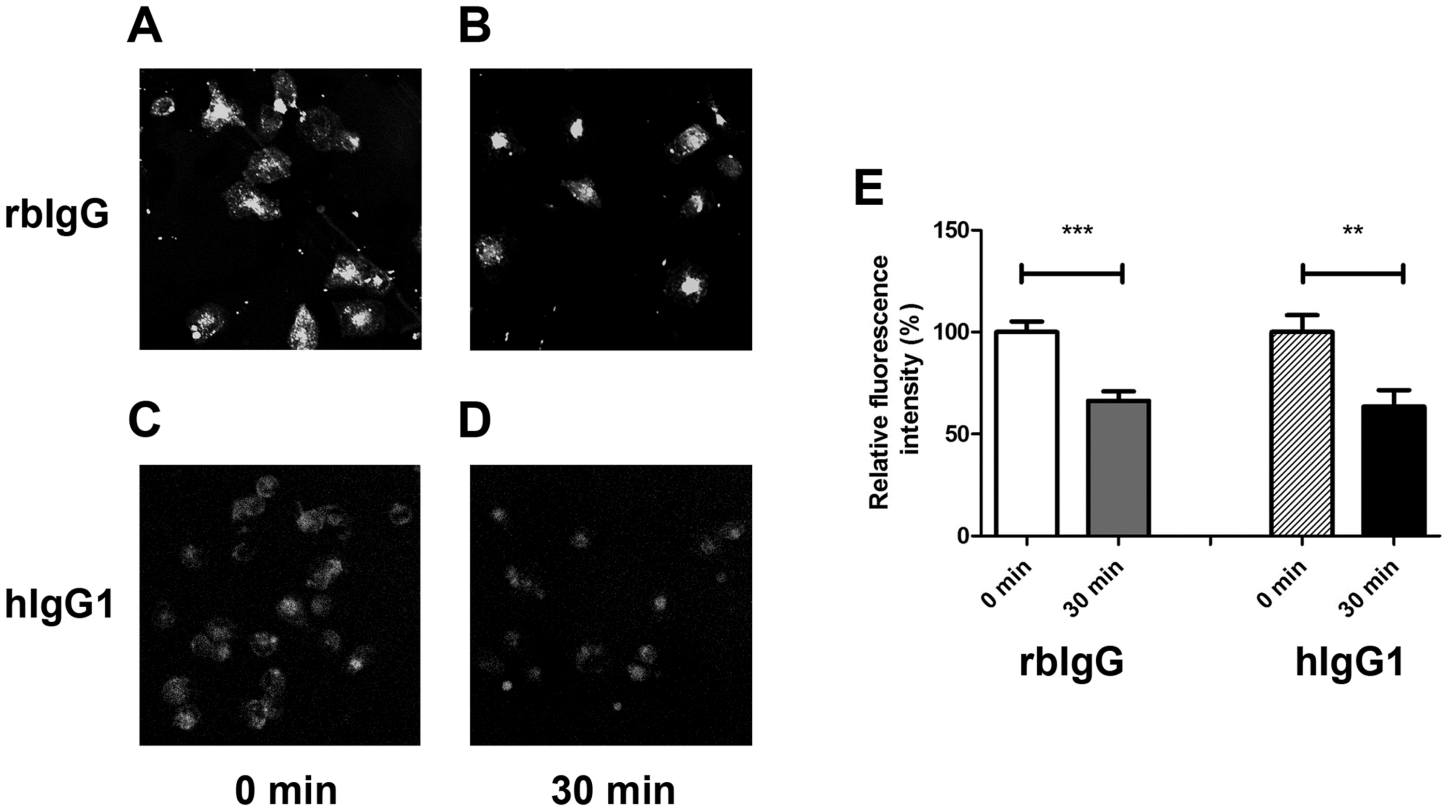
FcRn-mediated recycling studies of rbIgG and hIgG1 in rabbit peritoneal macrophages. Cells were pulsed with Alexa 488-conjugated rbIgG and hIgG1 for 20 minutes and chased for 30 minutes. Confocal images were collected at chase starting point (rbIgG on Panel A and hIgG1 on Panel C) and after 30 minutes (rbIgG on Panel B and hIgG1 on Panel D) and were processed using Fiji software [30]. Cells were drawn around, and the median level of pixel intensity were determined, and for comparison, relative fluorescent intensity of the cells were calculated in all cases (Panel E). Values shown are the means ± SEM (**, p< 0.01; ***, p<0.001).

Fig 5 shows that more fluorescent signal could be detected in rbIgG than that of hIgG1-labeled cells due to the different labeling efficiencies of IgGs. After 30 minutes of chase, significantly less rbIgG, as well as hIgG can be detected in macrophages and the relative fluorescence intensity decreased similarly to about 60% in both cases (Fig 5).

Our data shows no difference in the recycling rate of rbIgG and hIgG1 in rabbit macrophages supporting our SPR data, that rbFcRn can bind hIgG1 as effectively as rbIgG.

These data confirm the results of historic FcRn-related papers that reported 6.0 and 5.0 day long half-lives of rabbit and human 7S gamma globulin (which refers to IgG), respectively, in rabbits [24–26].

## Conclusions

Transplacental IgG transport in rabbits indicate that it occurs in a similar exposure time-window for offsprings as in humans, and rabbits as model, provide better statistical power. As we demonstrated by SPR experiments, as well as in FcRn-mediated recycling assay that rbFcRn similarly binds hIgG1, and preferentially binds hIgG2, hIgG3 and hIgG4 over rbIgG, rabbits can offer an alternative animal model to mouse and rats regarding IgG transport through the placenta. Our dataset also suggest that the hIgG producer rabbits would be fully functional regarding FcRn-mediated functions including maternal IgG transport, IgG protection and antigen presentation in case of antigen-IgG immune complexes.

## Supporting information

S1 Fig The raw, unadjusted source image corresponding to Fig 2A. (TIF)

S2 Fig The raw, unadjusted source image corresponding to Fig 2B. (TIF)
