# Supplementary figures and images for: Characterization of the interactions of rabbit neonatal Fc receptor (FcRn) with rabbit and human IgG isotypes
Source: PLoS One. 2017 Sep 28;12(9):e0185662. doi: 10.1371/journal.pone.0185662 (PMC5619814; doi:10.1371/journal.pone.0185662)

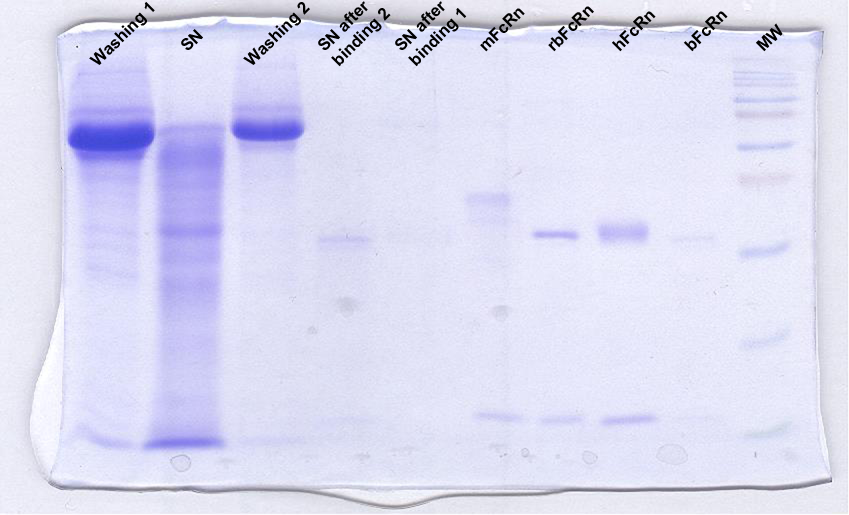

Supplement: S1 Fig — (TIF) [file pone.0185662.s001.tif]

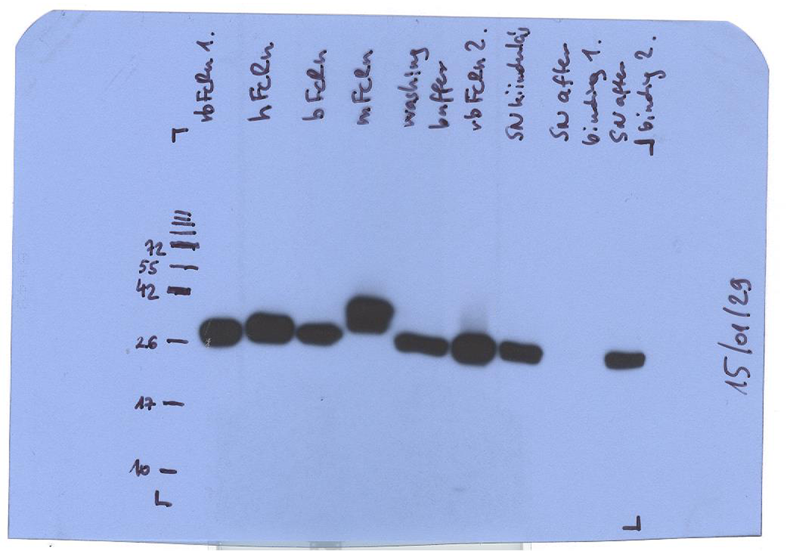

Supplement: S2 Fig — (TIF) [file pone.0185662.s002.tif]
